# *TAS1R2 rs35874116* Associations with Taste, Diet, and Health in an Italian Population

**DOI:** 10.3390/nu17020329

**Published:** 2025-01-17

**Authors:** Harry Stevens, Catherine Anna-Marie Graham, Maria Pina Concas, Francesco Piluso, Yiannis Mavrommatis, Alexandra King, Leta Pilic, Paolo Gasparini

**Affiliations:** 1 Department of Medical Sciences, University of Trieste, 34100 Trieste, Italy; francesco.piluso@llui.org (F.P.);; 2 Lake Lucerne Institute, 6354 Vitznau, Switzerland; cat.maher@llui.org (C.A.-M.G.);; 3 Cereneo Foundation—Center for Interdisciplinary Research, 6354 Vitznau, Switzerland; 4 Institute for Maternal and Child Health, IRCCS “Burlo Garofolo”, 34137 Trieste, Italy; 5 Faculty of Sport, Technology and Health Sciences, St Mary’s University Twickenham, London TW1 4SX, UKleta@optimysenutrition.com (L.P.); 6 MyHealthChecked PLC, Cardiff, CF24 5EA, UK; 7 Optimyse Nutrition Ltd., Radlett, WD7 9DJ, UK

**Keywords:** *TAS1R2*, taste, diet, health, BMI, nutrigenetics, SNP

## Abstract

Background/Objectives: The *TAS1R2* SNP *rs35874116* has previously been associated with sweet taste, diet, and health status, although never comprehensively in a single study. Also, associations between *TAS1R2* and sweet taste might be body mass index (BMI)-dependent. Therefore, this study aimed to conduct a comprehensive investigation of *rs35874116* and sweet taste intensity and liking, food liking, and diet and health status whilst considering BMI. Methods: Five-hundred and fifty-four participants were recruited. Linear regression models were used to explore *rs35874116* associations with sweet taste intensity and liking, food liking, and diet and health status. A secondary analysis stratified participants by BMI <25/≥25 kg/m^2^. Results: The *rs35874116* wildtype was associated with increased sweet taste intensity (*p* = 0.0345, B 1.29, SE 0.61) and liking (*p* = 0.021, B 0.25, SE 0.11). However, these associations only remained in BMI ≥25 individuals (intensity: *p* = 0.037, B 1.29, SE 0.61, liking: *p* = 0.008, B 0.46, SE 0.17). It was also associated with decreased diet quality (*p* = 0.03, B −0.27, SE 0.13) and reduced free sugar consumption but increased saturated fat consumption in BMI ≥25 individuals (free sugars: *p* = 0.0416, B −0.8, SE 0.38, saturated fat: *p* = 0.031, B 1.38, SE 0.62). There was no association with the mean liking score for sweet foods, although there were some associations with individual foods, which require further investigation. There were no associations with health status. Conclusions: This study revealed an association between the *rs35874116* wildtype and an increased intensity and liking of the sweet taste and a corresponding worse overall dietary quality. This study adds to previous evidence regarding how associations between *TAS1R2* and sweet taste are BMI-dependent.

## 1. Introduction

Suboptimal dietary choices are the leading cause of premature mortality, resulting in approximately 11 million deaths per year [1]. The most prominent influencer of dietary choice is taste [2]. Taste is a highly personal phenomenon which is largely influenced by genetics [3]. Humans have a natural preference for sweet tasting foods, as it signifies caloric density, but excessive consumption can lead to negative health outcomes [4]. The heritability of sweet taste preference has previously been estimated to be up to 54% [5].

Chemosensory cells are key moderators of taste response. Taste Receptor Type 1 Member 2 (*TAS1R2*), mostly expressed in taste buds, is a sweet taste receptor [6]. It encodes part of a G protein-coupled receptor which binds to both naturally occurring and artificial ‘sweet’ tasting molecules via a Venus flytrap module [6]. The *TAS1R2* missense single-nucleotide polymorphism (SNP) *rs35874116* causes an isoleucine > valine (T > C) conformational change on the ligand binding site of the protein, which may modify sweet taste reception [6,7].

Previous studies have investigated associations between *rs35874116* and sweet taste perception, diet, and health status. Taste or food liking, which is the pleasantness of the taste in the mouth, has never been explored. Also, previous *rs35874116* associations have not been explored comprehensively in a single study. Studies which have investigated *rs35874116* and sweet taste perception, measured by the subjective intensity of a given taste, or by taste discrimination tests, have not found consistent results [8,9,10]. But previous research has shown that sweet taste liking may have a bell-shaped response to sweet taste intensity [11]. Regarding dietary intake, liking of the sweet taste has been associated with increased liking, and in some cases, consumption of sweet foods [12,13,14,15], but this has not been explored in association with *rs35874116* genotype. The *rs35874116* wildtype ‘T’ allele has, however, been associated with greater consumption of carbohydrates, including typically non-sweet tasting carbohydrates such as fiber [7,16,17,18,19]. Previous studies have not consistently demonstrated that *rs35874116* variations are associated with health status [9,19,20,21]. However, some studies have found that the wildtype ‘T’ allele is associated with lower triglycerides, but greater levels of plasma glucose and insulin [22,23,24]. It is of vital importance that a comprehensive study combining *rs35874116,* taste, diet, and health be carried out, as it may be key for the development of personalized nutrition advice in future.

Sweet taste perception itself might be further modified by body mass index (BMI). Higher serum leptin levels, which are common in higher-BMI individuals, have been associated with greater sweet taste recognition thresholds [25]. However, BMI can cause leptin resistance. Leptin resistance might occur via several pathways, including downregulation of signal transduction, decreased histone deacetylase activity, increased levels of systemic inflammation, and reduced transport of leptin through the blood–brain barrier [26,27]. Eny et al. (2010) [7] demonstrates that the *rs35874116* TT genotype led to a higher consumption of sugars (total carbohydrates, fiber, sucrose, fructose, and glucose) in overweight individuals than individuals with the variant allele, whereas no difference was found in lean individuals. This finding suggests that increased sugar consumption amongst *rs35874116* T allele carriers is at least partly moderated by increased perceived sweet taste perception, but further research is needed to confirm this association.

To date, previous studies have investigated *rs35874116* associations with sweet taste intensity, dietary choices, and health status. However, no study has investigated its associations with sweet taste or food liking, or investigated all outcomes simultaneously on a single population, whilst also considering BMI. Therefore, due to the availability of a deeply genotypically and phenotypically characterized population, the aim of this study was to conduct a comprehensive investigation of *rs35874116* and sweet taste intensity and liking, food liking, and diet and health status on a single, Italian sample whilst considering BMI.

## 2. Materials and Methods

### 2.1. Recruitment

Five hundred and fifty-four participants were recruited from six genetically isolated villages in the Friuli-Venezia Giulia region of northeastern Italy. Participants were from the Italian Network of Genetic Isolates, a project which aims to uncover genetic and non-genetic causes of common diseases and traits. Participants were recruited via public advertisements through local authorities, televisions and newspapers, and local physicians and mailings. Meetings were organized to present the project and its aims. Screening was carried out from 2014 to 2015. No exclusion criteria were applied during the initial screening, but only participants aged 18–65 were included in the present study, and participants with missing data regarding age, sex, and genotype were excluded. Each enrolled participant was assigned a numerical code, and all de-identified and sensitive information was stored separately and only available to authorized personnel. All information stored in a database was password-protected and only available to authorized personnel. All participants provided informed consent. Ethical approval was granted by the Ethical Committee and Institutional Review Board of IRCCS “Burlo Garofolo” (under the univocal code Prot. CE/V-78, approval date: 6 August 2007). All investigations were carried out in accordance with the Declaration of Helsinki [28].

### 2.2. Demographic Information

Demographic information was collected via a self-administered standardized questionnaire, after an explanation by trained staff. Personnel were always available to answer participant questions. Demographic information collected included age (years) and sex (male/female/prefer not to say). A total of 554 participants aged 18–65 (45.4 ± 13 years, 57% female) were included in this study.

### 2.3. Taste Perception

Odors and auditory and visual distractions were controlled throughout screening using a dedicated, sensorially neutral room. Taste intensity and liking was measured by Labelled Magnitude Scale (LMS) and nine-point Likert scale (‘1’ indicated extremely dislike, ‘9’ indicated extremely like [29]), respectively, after explanation by trained staff, using filter paper representative of the sweet taste (sucrose 0.2 g/mL). This concentration was chosen because it is a medium concentration of a verified taste assessment methodology [30].

### 2.4. Food Liking

Food liking was assessed by a questionnaire which contained >100 foods and beverages (Supplementary Document S1) using the aforementioned 9-point Likert scale [29]. Food liking groups were created for ‘sweet’, ‘high saturated fat’ and ‘poor diet’ foods, and liking was recorded for each individual food, as well as the mean score for the overall group.

### 2.5. Diet

Diet outcomes were assessed by food frequency questionnaire (FFQ). Quality of diet was assessed by measuring whether Italian public health recommendations were met for four dietary metrics: total fat (20–35% total energy), saturated fat (<10% total energy), free sugars (<15% total energy), and fiber (>25 g) [31]. Calories (kcal) were also measured. A diet quality score from 0 (no recommendations met) to 4 (all recommendations met) was calculated for each participant.

### 2.6. Health Outcomes

#### 2.6.1. Anthropometrics

Height was measured to the nearest 0.25 cm using a stadiometer. Weight, BMI, and visceral fat were calculated using the Body Composition Analyzer (Tanita BC-420MA; Tanita, Tokyo, Japan). Waist-to-hip ratio was calculated by dividing waist by hip circumference (cm).

#### 2.6.2. Biomarkers

Biochemical outcomes assessed were serum glucose (mg/dL), triglycerides (mg/dL), total/HDL/LDL cholesterol (mg/dL), and insulin (mIU/L). Fasting blood samples were obtained early in the morning. Blood was tested on the same day or aliquoted and stored for further analysis. Biochemical analysis was performed with the Cobas 6000 Analyzer (Hoffmann-La Roche, Basel, Switzerland).

### 2.7. Genotyping

DNA was extracted from blood using phenol-chloroform extraction procedures. All individuals included in this study have been genotyped with Illumina 370 k high-density SNP array (Illumina Inc., San Diego, CA, USA). Genotypes were called with Illumina GenomeStudio and processed according to standard quality control procedures with the following criteria for inclusion: sample call rate ≥ 0.95, gender check, SNP call rate ≥ 0.95, Hardy–Weinberg Equilibrium *p*-value > 1 × 10−6, and minor allele frequency (MAF) ≥ 0.01. The SNP *rs35874116* was extracted and used in this study [32].

### 2.8. Statistical Analysis

A cross-sectional analysis was carried out. A Shapiro–Wilk test was used to assess the normality of continuous data. Pearson’s correlation was used to explore the association between sweet taste intensity and sweet taste liking. For differences in sample characteristics, continuous variables were assessed using a one-way ANOVA for normally distributed data, a Kruskal–Wallis test for non-normally distributed data, and a Chi-square test for genotype groups. *TAS1R2 rs35874116* genotypes were assigned values of 0, 1 or 2 (TT, TC and CC, respectively), and were analyzed in the additive genetic model to explore dose-dependent effects of alleles on phenotypic outcomes. The assigned values were used as predictors in linear regression models to explore *rs35874116* associations with sweet taste intensity and liking, food liking, and diet and health status, as this type of analysis allowed for the control of the covariates age and sex by their inclusion in the model. Analysis was then stratified by BMI <25/≥25 kg/m^2^ based on findings from Eny et al. (2010) [7] and analyses related to sweet taste were repeated. Suitability of food groupings was decided by Cronbach reliability test. Statistical analyses were performed using RStudio (version 2022.07.2 + 576) (www.R-project.org). The significance value was set at *p* < 0.05.

## 3. Results

### 3.1. Sample Characteristics

A total of 554 participants aged 18–65 were successfully genotyped for *rs35874116*. The genotype frequencies were TT—0.41, TC—0.46, CC—0.13. HWE was met (Chi-square *p* > 0.05). There were no significant differences between genotype groups in sex or age (Chi-square, Kruskal–Wallis > 0.05). Sample characteristics by overall population and genotype are displayed in Table 1.

### 3.2. TAS1R2 rs35874116 and Sweet Taste Intensity and Sweet Taste Liking

A significant positive correlation was found between sweet taste intensity and liking (*p* < 0.0001, Pearson 0.24, CI 0.16–0.33).

Linear regression models controlled for age and sex revealed significant positive associations between the *rs35874116* wildtype ‘T’ allele and sweet taste intensity (*p* = 0.0345, B 1.29, SE 0.61) and sweet taste liking (*p* = 0.021, B 0.25, SE 0.11) (Table 2).

After stratifying by BMI, significant associations between the *rs35874116* wildtype ‘T’ allele and sweet intensity and liking were only found in the BMI ≥ 25 group (BMI ≥ 25: N = 236, intensity: *p* = 0.037, B 1.29, SE 0.61, liking: *p* = 0.008, B 0.46, SE 0.17, BMI < 25: N = 307, intensity: *p* = 0.26, B 0.93, SE 0.83, liking: *p* = 0.35, B 0.13, SE 0.14).

### 3.3. Diet

Dietary data were collected for 126 participants (genotype frequencies among participants with dietary data TT—0.47, TC—0.40, CC—0.13). Linear regression models controlled for age and sex revealed significant positive associations between the *rs35874116* wildtype ‘T’ allele and decreased diet quality (*p* = 0.03, B −0.27, SE 0.13). There were no significant associations with the individual dietary components (total fat, saturated fat, free sugars, fiber) or total calories (Table 3).

After stratification by BMI, significant associations were found in the ≥25 group only. The *rs35874116* wildtype ‘T’ allele was associated with reduced free sugar consumption but increased saturated fat consumption (free sugars % of total energy: *p* = 0.0416, B −0.8, SE 0.38, saturated fat % of total energy: *p* = 0.031, B 1.38, SE 0.62, saturated fat kcal: *p* = 0.0296, B 63.2, SE 28.2). Associations with diet quality were no longer significant (*p* = 0.09, B −0.37, SE 0.21) (Table 4).

### 3.4. Food Liking

The food liking groups had Cronbach reliability scores > 0.5.

Linear regression models controlled for age and sex revealed no significant associations between *rs35874116* and the mean liking score for sweet, high saturated fat, or poor-diet-quality foods (Supplementary Document S2). Findings regarding the liking of individual foods and following stratification by BMI can be found in Supplementary Document S2.

### 3.5. Health Status

There were no significant associations between *rs35874116* and health status (Table 5).

## 4. Discussion

This study aimed to investigate associations between *TAS1R2 rs35874116* and (a) sweet taste intensity and liking, (b) food liking, (c) diet and (d) health status, whilst also considering BMI. We report that sweet taste intensity and liking were positively correlated, and that the *rs35874116* wildtype ‘T’ allele was associated with increased intensity and liking of the sweet taste. The *rs35874116* wildtype ‘T’ allele was not associated with the mean food liking score for any food groupings, but it was associated with some individual foods. The *rs35874116* wildtype ‘T’ allele was associated with worse overall diet quality, and a higher saturated fat but reduced free sugar consumption in individuals with a BMI ≥ 25. No notable associations were found between *rs35874116* and health status.

Regarding sweet taste intensity, previous evidence about the associations between *rs35874116* and sweet taste intensity is mixed, with some studies finding no associations [8,10], and another, Melis et al. (2022) [9], finding a higher intensity among those with the ‘CC’ genotype, contrasting our findings. However, Melis et al. (2022) only included 11 individuals with ‘CC’ genotype. Also, the delivery method of the sweet tasting vehicle differed from this study, highlighting the need for established ‘gold-standard’ methods to measure sweet taste. In support of our findings, previous pathway studies have demonstrated that the variant ‘C’ allele of *rs35874116* in *TAS1R2* limits binding of sweet molecules [6,7], which might explain why a higher intensity among *rs35874116* wildtype ‘T’ individuals was found. The novel investigation into *rs35874116* and sweet taste liking indicates that those with the wildtype ‘T’ allele had higher liking for the sweet taste. As sweet taste liking increases in accordance with intensity in the mid-range [11], our results are plausible. However, future research should continue to investigate the interrelatedness of sweet taste intensity and liking, as there is currently a dearth of literature.

Following stratification by BMI, we found that *rs35874116* associations with sweet taste intensity and liking only remained significant in individuals with BMI ≥ 25. BMI may modify sweet taste perception via an interaction with leptin [7]. Overweight individuals (BMI ≥ 25) often develop leptin resistance [26,27]. Although several pathways have been suggested for this, the likely mechanism for the association between high-BMI-associated leptin resistance and increased sweet taste intensity is a reduction in leptin-dependent signal transduction [26,27]. Leptin could reduce sweet taste intensity by causing outward K^+^ currents, which reduce cellular depolarization in response to ‘sweet’ compounds. Therefore, it can be hypothesized that overweight individuals perceive sweet taste as more intense. As sweet taste intensity was positively correlated with sweet taste liking, this can explain why there was also no association with sweet taste liking in individuals with BMI < 25. These results add to previous evidence regarding the link between BMI and sweet taste [7,25,26], but their connection requires further replication prior to consideration for personalized nutrition practice, as, to date, it has scarcely been explored. Also, some results garnered by stratifying by BMI may be limited by participant numbers, as there were only six individuals with the CC genotype in the BMI ≥ 25 group. Future studies should build upon these findings by continuing to explore whether sweet taste is dependent on *rs35874116* and BMI whilst ensuring an adequate sample size is achieved for taste-related outcomes in individuals with BMI ≥ 25.

*TAS1R2 rs35874116* was not associated with the sweet food liking group; however, the T allele was associated with liking of ‘marmalade’ in the BMI ≥ 25 group (Supplementary Document S2). Among the ‘sweet’ tasting foods included in the survey, standard marmalade products are amongst the highest for mono-/disaccharide-to-total carbohydrate and mono-/disaccharide-to-total calories ratio. As *TAS1R2* binds to typically sweet tasting molecules, including glucose, fructose and sucrose, which are high in marmalade, this association is consistent mechanistically. A possible reason that other sweet foods (Supplementary Document S2) did not score highly for liking was their increased fiber content. Longer-chain sugars might not bind to *TAS1R2* with the same affinity [6,33], thus lowering their perceived intensity. Therefore, this finding could align with results regarding the *rs35874116* wildtype ‘T’ allele and sweet taste intensity and liking in individuals with BMI ≥ 25. Future investigations should seek to include more high-mono-/disaccharide-content foods to confirm this finding, as, if true, those with the *rs35874116* wildtype might be at greater risk of overconsuming these sweet foods, and so this might shape personalized nutrition advice. However, the lack of other associations between *rs35874116* and food liking limits the practical applicability of this study, as it uncouples the results regarding single taste vehicles and complex foods and highlights the important distinction between an artificial lab setting and a real-world environment. Therefore, offering personalized nutrition advice regarding *rs35874116* and sweet food liking would not currently be appropriate, and future studies should seek to incorporate complex foods in their testing to maximize the practical applicability of findings. Additionally, the generalizability of these findings is limited by the inclusion of a singular, homogenous cohort. Future studies should seek to investigate additional populations to establish the replicability of these findings.

A large proportion of research has endeavored to understand the mechanisms and influences of sweet taste; however, comparatively little research has assessed this influence on dietary intake. We found that the *rs35874116* wildtype was associated with a worse overall diet quality and increased saturated fat consumption while decreasing free sugar consumption in individuals with BMI ≥ 25 (Table 3 and Table 4). Whilst the association with worse dietary quality did not reach statistical significance in individuals BMI ≥ 25, the trend remained. The leading contributor to worse overall dietary quality among *rs35874116* wildtype carriers in individuals with BMI ≥ 25 appears to be the replacement of dietary carbohydrates with fats. However, previous research has found evidence to the contrary, as generally, *rs35874116* wildtype carriers consume more carbohydrates [7,16,17,18,19]. Our study did not replicate any of these findings. However, many of these studies have assessed younger populations, who may have different dietary compositions and desires compared to the present study population. In fact, in the present study, after stratification by BMI, *rs35874116* wildtype individuals with BMI ≥ 25 consumed fewer free sugars (Table 4). A previous study associated the *rs35874116* wildtype with reduced sugar consumption due to sensory-specific satiety [10], which is worth consideration as the *rs35874116* wildtype ‘T’ allele was associated with higher sweet taste intensity. Future studies should seek to investigate possible behavioral links between reducing carbohydrate consumption and increasing fat consumption, as worse diet quality and increased saturated fat consumption could lead to deleterious health consequences. If associations between *TAS1R2* and over-consumption of carbohydrates and/or fats can be established, this could be a key area for personalized nutrition advice in future. However, the current overall lack of credible links between taste and diet is a limitation of the practical applicability of present study, and personalized nutrition advice would not be appropriate. It also must be noted that taste is only a partial reason for food choice, and other reasons, including health, cost, time, and social relationships, must always be considered [34,35]. Additionally, gathering of dietary data could have been optimized by using a multiple-day food record or multiple method analysis rather than an FFQ. FFQs are liable to inaccuracies, including omission/addition of foods and poor self-assessment of the quantity of foods consumed, often due to social desirability bias [36,37]. Multiple-day food records or multiple method analysis has been proven to reduce these limitations, and future studies should seek to employ them to improve the accuracy of associations between *TAS1R2* and diet [36,37].

To date, very little research has investigated the association between sweet taste and health outcomes. Relationships between *rs35874116* and health status previously described, such as lower triglycerides and greater levels of plasma glucose and insulin [22,23,24], were not replicated. However, the studies that found associations with plasma glucose and insulin largely investigated extra-oral tissues and were not directly linked to any diet or lifestyle changes associated with *TAS1R2* and so are limited in their comparability to the present study [23,24]. The previous study that found an association between the *rs35874116* wildtype and decreased triglycerides found this in accordance with a reduced consumption of total carbohydrates, fiber, cereal and vegetables, which was not the case in the present study [22]. The presents study’s findings regarding decreased free sugar consumption alone did not produce the same associations with increased triglycerides. Future dietary intervention studies, in particular comparing high-fat to high-carbohydrate diets, would help to confirm any effects of *rs35874116* on health. Additionally, longitudinal studies are required to assess the effects of *rs35874116* on health over time. As current studies that link *TAS1R2* to health outcomes are scarce, future studies should seek to include this in their investigations to better explain the links between *rs35874116*, taste, diet and health.

## 5. Conclusions

To our knowledge, this is the first study to investigate the associations between *TAS1R2 rs35874116* and (a) sweet taste intensity and liking, (b) food liking, (c) diet and (d) health status, whilst also considering BMI. Here, we demonstrate an association between the *rs35874116* wildtype ‘T’ allele and an increased intensity and liking of the sweet taste and a corresponding worse overall dietary quality. These results further elucidate links between taste perception and real-world dietary choices. This study also added to previous literature regarding how *rs35874116* is associated with different taste- and diet-related outcomes depending on BMI. Also, this study found that the *rs35874116* wildtype ‘T’ allele might be associated with increased liking of high-mono-/disaccharide-content sweet foods. However, there were no associations between *rs35874116* and health outcomes. As associations between *rs35874116* and food liking have scarcely been investigated, and previous studies have found associations between *rs35874116* and health outcomes, future studies should continue to explore these outcomes to increase the practical applicability of current findings.

## Figures and Tables

**Table 1:** Sample characteristics, including Taste Receptor Type 1 Member 2 (*TAS1R2*) *rs35874116* genotype frequencies and Hardy–Weinberg Equilibrium (HWE) significance value, sex (number and % female), age (years, mean ± SD), and sample characteristic comparisons.

	Overall	*TAS1R2 rs35874116* Genotype TT	*TAS1R2 rs35874116* Genotype TC	*TAS1R2 rs35874116* Genotype CC	Significance Value
N (Frequency)	554	227 (0.41)	255 (0.46)	72 (0.13)	*p* > 0.05
Sex	316 (57%)	137 (60%)	141 (55%)	38 (53%)	*p* > 0.05
Age	45.4 ± 13	45.2 ± 12.8	45.1 ± 12.9	47.1 ± 13.7	*p* > 0.05

**Table 2:** Summary statistics for the association between *TAS1R2 rs35874116* and sweet taste intensity (mean ± SD) (LMS) and sweet taste liking (mean ± SD) (Likert scale), as assessed by linear models controlled for age and sex.

	*TAS1R2 rs35874116* Genotype TT	*TAS1R2 rs35874116* Genotype TC	*TAS1R2 rs35874116* Genotype CC	Significance Value
Sweet Taste Intensity	13.9 ± 10.07	13.17 ± 9.61	10.79 ± 7.51	*p* = 0.0345
Sweet Taste Liking	6.12 ± 1.67	5.94 ± 1.6	5.57 ± 1.73	*p* = 0.021

**Table 3:** Summary statistics for the association between *TAS1R2 rs35874116* and total fat (mean kcal ± SD), total fat % of total kcal (mean ± SD), saturated fat (mean kcal ± SD), saturated fat % of total kcal (mean ± SD), free sugars (mean kcal ± SD), free sugars % of total kcal (mean ± SD), fiber (mean g ± SD), total calories (mean ± SD) and diet quality score as assessed by linear models controlled for age and sex.

	*TAS1R2 rs35874116* Genotype TT	*TAS1R2 rs35874116* Genotype TC	*TAS1R2 rs35874116* Genotype CC	Significance Value
Total Fat	1038.73 ± 304.86	971.67 ± 242.51	1003.22 ± 200.72	*p* > 0.05
Total Fat % Of Total kcal	36 ± 5.3	35.45 ± 5.05	34.4 ± 6.87	*p* > 0.05
Saturated Fat	317.1 ± 134.9	269.77 ± 88.94	285.38 ± 105.3	*p* > 0.05
Saturated Fat % Of Total kcal	10.82 ± 3.07	9.77 ± 2.46	9.76 ± 4.05	*p* > 0.05
Free Sugars	348.49 ± 127.27	336.16 ± 109.73	376.31 ± 125.69	*p* > 0.05
Free Sugars % Of Total kcal	11.82 ± 1.64	12.06 ± 1.84	12.33 ± 2.01	*p* > 0.05
Fibre	22.01 ± 7.28	21.64 ± 6.98	23.93 ± 7.62	*p* > 0.05
Total Calories	2953.65 ± 1005.96	2788.7 ± 769.27	3006.12 ± 738.73	*p* > 0.05
Diet Quality Score	2	2.29	2.71	*p* = 0.03

**Table 4:** Summary statistics for the association between *TAS1R2 rs35874116* and total fat (mean kcal ± SD), total fat % of total kcal (mean ± SD), saturated fat (mean kcal ± SD), saturated fat % of total kcal (mean ± SD), free sugars (mean kcal ± SD), free sugars % of total kcal (mean ± SD), fiber (mean g ± SD), total calories (mean ± SD) and diet quality score in body mass index (BMI) (kg/m^2^) ≥25 individuals as assessed by linear models controlled for age and sex.

	*TAS1R2 rs35874116* Genotype TT	*TAS1R2 rs35874116* Genotype TC	*TAS1R2 rs35874116* Genotype CC	Significance Value
Total Fat	1104.98 ± 397.92	981.83 ± 260.07	1059.38 ± 292.99	*p* > 0.05
Total Fat % Of Total kcal	35.81 ± 6.38	34.93 ± 5.31	34.89 ± 5.44	*p* > 0.05
Saturated Fat	367.39 ± 171.03	278.03 ± 89.09	272.6 ± 134.17	*p* = 0.03
Saturated Fat % Of Total kcal	11.68 ± 3.45	9.97 ± 2.78	8.61 ± 2.56	*p* = 0.031
Free Sugars	355.9 ± 153.93	344.28 ± 110.9	382.82 ± 130.18	*p* > 0.05
Free Sugars % Of Total kcal	11.21 ± 1.73	12.16 ± 2.06	12.33 ± 1.46	*p* = 0.042
Fibre	22.45 ± 9.69	21.27 ± 7.09	24.89 ± 8.22	*p* > 0.05
Total Calories	3200.96 ± 1332.59	2859.14 ± 822.16	3076.49 ± 860.8	*p* > 0.05
Diet Quality Score	1.76	2.32	2.67	*p* = 0.09

**Table 5:** Summary statistics for the association between *TAS1R2 rs35874116* and serum glucose (mg/dL mean ± SD), triglycerides (mg/dL mean ± SD), total cholesterol (mg/dL mean ± SD), high-density lipoprotein (HDL) cholesterol (mg/dL Mean ± SD), low-density lipoprotein (LDL) cholesterol (mg/dL mean ± SD), insulin (mIU/L mean ± SD), waist–hip ratio (waist/hip circumference), visceral fat (1–59 mean ± SD) and BMI (kg/m^2^) as assessed by linear models controlled for age and sex.

	*TAS1R2 rs35874116* Genotype TT	*TAS1R2 rs35874116* Genotype TC	*TAS1R2 rs35874116* Genotype CC	Significance Value
Serum Glucose	93.39 ± 25.5 * (219)	90.74 ± 19.17 (247)	91.76 ± 11.27	*p* > 0.05
Triglycerides	106.62 ± 73.06 (219)	106.31 ± 58.77 (246)	123.96 ± 89.92	*p* > 0.05
Total Cholesterol	210.99 ± 41.09 (219)	212.98 ± 44.01 (247)	219.58 ± 51.34	*p* > 0.05
HDL Cholesterol	63.72 ± 16.53 (219)	63.09 ± 17 (247)	60.29 ± 17.88	*p* > 0.05
LDL Cholesterol	125.94 ± 36.72 (219)	128.2 ± 40.39 (247)	134.5 ± 40.65	*p* > 0.05
Insulin	9.62 ± 7.28 (219)	9.26 ± 5.34 (243)	11.12 ± 8 (67)	*p* > 0.05
WHR	0.88 ± 0.1 (122)	0.9 ± 0.09 (145)	0.91 ± 0.12 (41)	*p* > 0.05
Visceral Fat	6.6 ± 4.18 (211)	7.38 ± 4.69 (240)	8.45 ± 5.65 (69)	*p* > 0.05
BMI	25.05 ± 4.92 (222)	24.91 ± 4.4 (249)	24.87 ± 4.73	*p* > 0.05

* Where there are missing data, number of participants with available data is stated.

## Data Availability

A subset of the data is already available in the European Genome-phenome Archive (EGA) at the following links. FVG cohort: BAM files https://www.ebi.ac.uk/ega/studies/EGAS00001000252 (accessed on 3 November 2023); sample list, vcf files https://www.ebi.ac.uk/ega/studies/EGAS00001001597 (accessed on 3 November 2023); https://www.ebi.ac.uk/ega/datasets/EGAD00001002729 (accessed on 3 November 2023). Other data presented in this study are available on request from the corresponding author.

## Supplementary Materials

The following supporting information can be downloaded at: https://www.mdpi.com/article/10.3390/nu17020329/s1, Document S1: Supplementary_Document_1. Document S2: Supplementary_Document_2.
